# Severe hepatitis with autoimmune features following a HHV-6: a case report

**DOI:** 10.1186/1757-1626-1-110

**Published:** 2008-08-18

**Authors:** Pierfrancesco Grima, Roberto Chiavaroli, Paola Calabrese, Paolo Tundo, Piero Grima

**Affiliations:** 1 Division of Infectious Disease, "Santa Caterina Novella" Hospital, Galatina, Italy

## Abstract

HHV-6 has been identified as the aetiologic agent of exanthem subitum in infants and an acute febrile illness in young children. HHV-6 probably remains latent in the body after the primary infection and it reactivates upon host immunosuppression in a manner similar to other human herpes viruses. Primary HHV-6 infection in adults is very rare and it is not clear whether disease manifestations are similar to those observed in children.

We report the case of acute hepatitis in a 18-year-old immunocompetent woman presenting with sever jaundice and liver dysfunction. Serum immunoglobulin levels were elevated (3.8 gr/dl) with a titre of anti nucleus antibody of 1:640. Serological data demonstrated the presence of IgM antibodies against human herpesvirus-6 in the serum and of viral DNA on liver biopsy by real time quantitative polymerase chain reaction, with a viral load of 280 genomes/10^6^ of cellular genomes. No other etiologic agents were found to induce hepatitis and the patient was diagnosed as having HHV-6 triggered autoimmune acute hepatitis.

## Introduction

Human herpesvirus 6 (HHV-6) was first isolated from patients with the acquired immunodeficiency syndrome or lymphoproliferative diseases and was named human B lymphotropic virus [1]. HHV-6 has been identified as the etiologic agent of exanthema subitum in infants [2] and an acute febrile illness in young children [3]. Most people are seropositive for HHV-6 by the age of three years [4]. HHV-6 also produces latent or chronic infections [5] and is occasionally reactivated in immunocompromised hosts [1,6]. Furthermore, HHV-6 has been implicated in several diseases in immunocompetent adults, including Kikuchi's lymphadenitis [7] and an infectious mononucleosis-like syndrome that is negative for Epstein-Barr virus and cytomegalovirus [8]. We describe the immunopathological and clinical features of a severe acute hepatitis in a 18-year-old woman that was probably caused by a primary infection with HHV-6.

## Case presentation

A 18-year-old woman was admitted to S.Caterina Novella Hospital on October 10, 2006, with a fifteenday history of flu-like syndrome. She had been healthy and had a history of self-limiting viral infections including measles and rubella in childhood. Physical examination revealed left cervical lymphadenopathy, splenomegaly and sever jaundice. Abnormal laboratory findings included a white blood cell count of 4.9 × 10^9^/L (3% atypical lymphocytes) with large granular cells and anisocytosy in peripheral smear.

Liver dysfunction was seen, with an increase in the levels of aspartate aminotransferase (1515 IU/l), alanine aminotransferase (1658 IU/l), lactate dehydrogenase (1080 IU/l) and total bilirubin (18.6 mg/dl). Prothrombin time was 28%. Serum immunoglobulin levels were elevated (3.8 gr/dl) with a titre of anti nucleus antibody (ANA) of 1:640. No antibodies against human immunodeficiency virus (HIV), hepatitis C virus (HCV), Hepatitis B virus (HBV), Cytomegalovirus (CMV), Epstein Barr Virus (EBV) were detected. However anti-HHV-6 antibody (IgG and IgM) were detected with IgM index of 3.2 (cut off for positive control > 1.1). A diagnosis of hepatic failure was made, and liver biopsy was performed during the acute stage. Histologic examination showed moderate infiltration of atypical lymphoid cells and diffuse focal vacuolar degeneration of hepatocytes. The infiltrating lymphocytes were positive for CD3, CD4, and CD8, but negative for CD20. The presence of HHV-6 DNA was shown in liver tissue by polymerase chain reaction (PCR) with a viral load of 280 genomes/10^6 ^of cellular genomes, suggesting active viral replication in the hepatocytes. Methylprednisolone was administered for three weeks beginning on the seventh day of hospitalization with dosage of 25 mg every twelve hours. The jaundice, lymphadenopathy and splenomegaly gradually disappeared and patient was sent home on the 35th hospital day with a normal hepatic function and no clinical sequelae. At 2 months HHV6 IgM antibodies decreased and disappeared after 3 months.

## Discussion

Our data indicate that HHV-6 was the cause of our patient's acute illness. Serologic studies excluded the possibility of active infection by HCV, HBV or other human herpesviruses such CMV and EBV. The presence of HHV-6 IgM antibodies shortly after the onset of liver disease and positive ANA titres suggest that HHV6 or an autoimmune disease may also be involved in the pathogenesis. HHV-6 is a CD4 lymphotropic virus isolated from T-cells cultures derived from the blood of subjects HIV+ [1]. Infection by HHV6 is rapidly controlled by the host immune response, and the virus established a state of latency. Primary infection occurs mostly in early childhood and only rarely in adults, in whom the prevalence of anti-HHV6 IgG is more than 90% [3]. Symptomatic infection is characterized by fever, skin rash (exanthema subitum), sometimes associated with mild respiratory illness, leukopenia and atypical lymphocytosis [3,10]. Recovery is usually rapid and benign, although a more severe course with meningitis, encephalitis, myocarditis or hepatitis, variable from mild hepatitis to fulminant liver failure, has been described [3]. HHV6 has also been associated with interstitial pneumonia and encephalitis in immunocompromised patients. [11] We assume that HHV-6 caused the initial clinical manifestations but an humoral virus-triggered autoimmune reaction, indicated by the positive ANA titre, responding to immunosuppression induced hepatic damage. Manifestations of autoimmune hepatitis have been described repeatedly after infection with hepatitis A, B and C as well as with herpes viruses, namely HSV1, EBV and HHV6 [12,13].

## Conclusion

Autoantibodies may be triggered by a virus specific mechanism to evade immune responses called 'molecular mimicry', when domains on viral proteins closely resembling human self-epitopes are generated [14,15]. Thus, we believe that in addition to causing exanthem subitum in infants and a febrile illness in children, HHV-6 type B can cause an acute and potentially fulminant hepatitis in adults with an autoimmune pathogenetic mechanism.

## Competing interests

The authors declare that they have no competing interests.

## Authors' contributions

PGjr acquisited data and made analysis and interpretation of data, PC assessed ultrasonographic examination, RC assessed liver biopsy, PG analized tissue molecular test and helped to draft the manuscript. All authors read and approved the final manuscript. Informed written consent was received from the patient for publication of the manuscript.
